# Intergroup, Intragroup, and Changing Best Friendships: Implications for Prejudice and Attitudes Toward Integration in Ethnic Majority and Minority Adolescents

**DOI:** 10.1007/s10964-026-02354-5

**Published:** 2026-04-15

**Authors:** Hao Yang, Beatrice Bobba, Francesca Prati, Elisabetta Crocetti

**Affiliations:** 1 Alma Mater Studiorum University of Bologna, Bologna, Italy; 2 Utrecht University, Utrecht, the Netherlands

**Keywords:** Multicultural society, Intergroup contact, Types of best friendships, Prejudice, Attitudes toward integration, Adolescence

## Abstract

Intergroup friendships are crucial to promote positive intergroup relations. However, it is less clear whether intragroup and intergroup best friendships are stable in adolescence and how they are intertwined with the development of prejudice and attitudes toward integration. The present study addressed these questions among ethnic majority and minority adolescents in Italy. Participants were 1,227 youth (*M*_age_ = 15.67, *SD* = 1.20, 47.34% female), of whom 82.56% had an ethnic majority Italian background and 17.44% an ethnic minority background. Ethnic majority adolescents predominantly maintained stable intragroup best friendships, whereas ethnic minority youth were more likely to have stable intergroup best friendships or to change their friendship type. Best friendship type and stability moderated the development of prejudice but not of attitudes toward integration. This study highlights that leveraging the potential of intergroup friendships can be an effective way to reduce heinous forms of prejudice toward individuals with a different cultural background, but may not be enough to foster more abstract inclusive attitudes.

## Introduction

In current ethnically and culturally diverse societies (International Organization for Migration, 2024), adolescents’ daily interactions take place in increasingly multicultural environments, and contact with peers from different ethnic groups is part of daily life (Meissner, 2019). Despite these opportunities for intercultural learning and mutual understanding, persistent patterns of intergroup segregation indicate that prejudice between ethnic majority and minority groups remains widespread (Crocetti et al., 2021). Best friendship ties among adolescents from different ethnic backgrounds may be an effective catalyst for promoting positive intergroup attitudes (Bowker & Weingarten, 2022; Wölfer et al., 2016). However, few studies have investigated the stability of different types of best friendships (i.e., intragroup, intergroup) in adolescence, and whether they influence the development of intergroup attitudes during this life phase. This longitudinal study examines the extent to which intragroup and intergroup best friendships remain stable throughout adolescence, and how such stability is intertwined with the development of prejudice and attitudes toward integration among ethnic majority (i.e., youth born in Italy from two Italian parents) and ethnic minority adolescents (i.e., youth living or born in Italy with at least one parent born abroad) in the Italian context.

### Best Friendship Ties in Multicultural Societies

Friendship is a close interpersonal relationship characterized by warmth and mutual support (Ajrouch et al., 2024; Heshmati et al., 2021). Especially in adolescence, friendship becomes a crucial context for personal growth and development (Rubin et al., 2015; Schwartz-Mette et al., 2020). Notably, compared to other types of friendship, *best friend relationships* are more intimate, involve higher levels of self-disclosure, trust, and loyalty, and imply a certain degree of exclusivity (Berndt, 2002; Langheit & Poulin, 2024). As such, best friendships might more strongly impact adolescents’ thoughts and feelings about the self and others, their behaviors, and adjustment (Bowker & Weingarten, 2022).

However, the formation of best friendships in multicultural contexts is not always straightforward. On the one hand, individuals usually display a tendency to establish friendship ties with others who are similar to them based on certain characteristics (e.g., sex, age, ethnicity). Such homophily principle (McPherson et al., 2001) might facilitate the formation of intragroup friendships and inhibit intergroup friendships. Along this line, prior research has highlighted that adolescents are more likely to form friendships with and choose playmates from their own ethnic ingroup, rather than the outgroup (Kelleghan et al., 2019; Munniksma & Juvonen, 2012). Additionally, adolescents were significantly less likely to nominate intergroup peers as ‘best friends’ compared to intragroup peers (Reynolds, 2007).

On the other hand, people are more likely to form relationships with those who are physically or contextually close to them (Mouw & Entwisle, 2006). Thus, the propinquity principle might support the formation of intergroup friendships in multicultural contexts. This idea aligns with the contact hypothesis (Allport, 1954; Pettigrew & Tropp, 2006), which argues that intergroup contact fosters familiarity and positive attitudes between individuals from different ethnic backgrounds, ultimately promoting intergroup friendships. Supporting this, a substantial body of research has shown that adolescents living in more ethnically diverse environments (e.g., schools or communities) are more likely to form intergroup friendships (Chan & Benner, 2025; Devine et al., 2024; Quillian & Campbell, 2003).

Beyond their initial formation, the stability of best friendships represents a critical dimension of relationship development (Poulin & Chan, 2010), and it might have more long-lasting implications for interpersonal adjustment and intergroup attitudes. Research has found that, compared with transient or frequently changing relationships, enduring friendships are more strongly associated with adolescents’ attitudes and behavioral adjustment (Bowker & Weingarten, 2022). High-quality, stable friendships provide deeper intimacy and emotional disclosure, and sustained opportunities for discussion and conflict resolution (Costello et al., 2024; Öztürk & Sumbas, 2023). As such, stability amplifies the positive implications of friendship, with stable (best) friendships exerting a stronger and long-lasting impact on adolescents’ development and well-being (Poulin & Chan, 2010).

However, during adolescence, friendships are often highly unstable (Bowker & Weingarten, 2022; Meter & Card, 2016), as young people continually explore and adjust their social networks to meet developmental needs (Benner, 2011). This instability is especially pronounced in intergroup friendships (Jugert et al., 2013; Lessard et al., 2019; Oczlon et al., 2023), which may hinder the development of intergroup intimacy (Aboud & Sankar, 2007; Rude & Herda, 2010) and, consequently, further influence the development of positive intergroup attitudes. Nevertheless, previous research on the stability of intergroup friendships has largely focused on general friendships, and little is known about how the stability of best friendships specifically affects the development of intergroup attitudes.

### Prejudice and Attitudes toward Integration in Adolescence: The Role of Best Friendship Ties

In multicultural societies, individuals can show different levels of negative (i.e., prejudice) and positive (i.e., attitudes toward integration) intergroup attitudes. Specifically, *prejudice* entails a set of negative beliefs, emotions, and behaviors toward individuals because of their different ethnic backgrounds (Allport, 1954; Brown, 2011). *Attitudes toward integration*, by contrast, can be reflected in adolescents’ support for policies that facilitate immigrants’ inclusion in the host society (Maratia et al., 2024).

In adolescence, youth develop a clearer sense of themselves and others (Meeus, 2019) and progressively consolidate their social and political views (Crocetti et al., 2021), such as prejudice and attitudes toward integration. Although conceptually distinct, both negative and positive intergroup attitudes can be shaped by overlapping psychological mechanisms (e.g., identity; Bobba et al., 2023) and contextual influences (e.g., family processes; Bobba et al., 2024a, Maratia & Crocetti, 2024). Among these, forming and maintaining best friendship ties with a member of the outgroup might reduce prejudice and foster attitudes toward integration (Killen et al., 2021; Wölfer et al., 2016). Intergroup friendships were found to be associated with more positive intergroup attitudes (Chen & Graham, 2015) but also to contribute to significant reductions in prejudice over time (Lutterbach & Beelmann, 2025; Wölfer et al., 2016). Ethnic majority German adolescents who maintained intergroup friendships over a three-year period showed significant reductions in ethnic prejudice during the same timespan (Titzmann et al., 2015). Similarly, the stability of intergroup friendships was found to contribute to greater positive intergroup attitudes, above and beyond the effect of the total number of intergroup friendships adolescents had (Rastogi & Juvonen, 2019).

Despite these contributions, several open questions remain. First, existing research has primarily focused on the role of intergroup friendships in reducing prejudice (Davies et al., 2011; Pettigrew & Tropp, 2008), while their potential in promoting the development of positive attitudes (i.e., favoring integration) remains less explored. Second, most studies (e.g., Lutterbach & Beelmann, 2025; Titzmann et al., 2015) have examined these dynamics only from the perspective of ethnic majority youth, thus without accounting for the experience of ethnic minority adolescents. Last, limited attention (but see Rastogi & Juvonen, 2019; Titzmann et al., 2015) has been paid to the stability of intergroup (best) friendships and how it contributes to changes in intergroup attitudes over time. Adopting an intergroup and longitudinal perspective is crucial to shed light on the development of best friendship ties in multicultural contexts and their implications for adolescent perceptions of and adjustment to ethnic and cultural diversity.

## Current Study

In recent years, Italy has emerged as a major destination for migrants in Europe, making ethnic and cultural diversity a normative context for adolescent socialization. Although intergroup friendship is an important driving force for reducing negative intergroup attitudes, limited attention has been paid to the stability of intragroup and intergroup best friendship ties in multicultural contexts and their implications for the development of both negative (i.e., prejudice) and positive attitudes (i.e., attitudes toward integration). The present study aimed to fill these research gaps by considering both ethnic majority and minority adolescents. First, it examined the prevalence of stable intragroup and intergroup best friendships among both groups. Drawing on the homophily principle and prior research, stable intragroup best friendships were expected to be more common among all youth (Hypothesis 1). Second, this study investigated whether adolescents with different types of best friendships (i.e., stable intragroup, stable intergroup, and unstable) would display unique developmental trajectories in their negative (i.e., prejudice) and positive (i.e., attitudes toward integration) intergroup attitudes. In line with the intergroup contact hypothesis and prior findings, ethnic majority and minority adolescents with stable intergroup friendships were expected to display significant decreases in prejudice (Hypothesis 2a) and increases in positive attitudes toward integration (Hypothesis 2b). For these analyses, the hypotheses were tested while accounting for adolescents’ structural opportunities for intergroup contact in the classroom.

## Methods

### Participants

Data for the current study were drawn from the cohort-sequential longitudinal project IDENTITIES “Managing identities in diverse societies: A developmental intergroup perspective with adolescents”, which followed adolescents from 15 high schools located in the North-East part of Italy (i.e., the Emilia-Romagna region), together with their parents and teachers. The present study relies on adolescents’ data collected across seven waves from January/February 2022 to January/February 2024. A total of 1,547 youth completed at least one of the study assessments. For the current study, nine international adopted children were not included (due to difficult classification as majority/minority). Furthermore, given the focus on stability of best friendships, only adolescents who provided information about their best friend’s nationality at least twice were included.

This resulted in a final analytical sample of 1,227 adolescents (*M*_age_ = 15.67, *SD* = 1.20; range: 13.79–20.04 years; 47.34% females) attending the 1^st^ (51.75%) and 3^rd^ (48.25%) year of high school at the beginning of the study (i.e., January/February 2022). The sample included two groups of youth based on their ethnic background. Specifically, 1,013 adolescents (*M*_age_ = 15.63, *SD* = 1.16, range: 13.79–20.04 years; 45.99% females; 51.33% 1^st^ year students) had an ethnic majority Italian background (i.e., their parents were both born in Italy), while the remaining 214 adolescents (*M*_age_ = 15.89, *SD* = 1.37, range: 13.87–19.40 years; 53.77% females; 53.74% 1^st^ year students) had an ethnic minority background (i.e., at least one of their parents was born outside Italy). Within the subsample of ethnic minority adolescents, a quarter (26.20%) were first-generation (i.e., born outside Italy) and almost three-quarters (73.80%) were second-generation youth who were born in Italy. Participants were drawn from multiethnic classrooms in which the average proportion of ethnic minority students was 17.16% (range = 0.00%–50.00%). The distribution of ethnic majority and minority youth in the study fully aligns with the school demographics of the Emilia-Romagna region, where ethnic minority students account for 18.4% of the student population (Ministero della Pubblica Istruzione, 2024). At baseline, adolescents reported that about half (49.14%) of their mothers had a medium educational level (i.e., high school diploma), followed by those (32.97%) with a high (i.e., university degree or higher) and a few (17.89%) with a low (i.e., up to middle school diploma) educational level. Regarding fathers, approximately half of the participants (49.23%) indicated a medium educational level, while the remaining participants reported low (27.02%) or high (23.75%) educational levels. Further details about participants’ demographics (e.g., country of origin of ethnic minority youth) and group comparisons are available in the Supplemental Materials.

Most participating adolescents (84.27%) completed three or more assessments. Within each assessment, the completion rate ranged from 59.49% at T6 to 77.42% at T2, and missingness was mostly due to participants not filling out the questionnaire because they were not in school on the day of data collection. The Little’s (1988) Missing Completely at Random (MCAR) test yielded a normed χ^2^ (χ^2^/df = 9612.84/7539) of 1.27, indicating that data were likely missing completely at random. Therefore, the total sample of 1,227 adolescents was included in the analyses, and missing data were handled with the Full Information Maximum Likelihood (FIML) procedure available in M*plus* (Kelloway, 2015), which is recommended in social science research (Enders, 2013). In addition, to evaluate the potential impact of sample attrition and ensure the robustness of the findings, a series of attrition analyses were conducted (see Tables S1 and S2). Results indicated that although some selective attrition occurred, the effects were of small magnitude and unlikely to confound the primary results.

### Procedure

This study was approved by the Ethics Committee of the Alma Mater Studiorum University of Bologna (Italy) as part of the IDENTITIES project, from which the current data were drawn. Schools were selected through a stratified (by school track and level of urbanization) randomized method. Specifically, school selection encompassed different educational tracks (i.e., academic, technical, and vocational) as well as areas with varying levels of urbanization (i.e., highly urbanized, moderately urbanized, and rural regions). In addition, the number of schools selected from each area (i.e., province) was determined proportionally based on the population size of that given district. This strategy ensured the selection of a representative sample of adolescents.

School principals were approached to present the project. Upon their approval, the study was presented to students and their parents, who also received written detailed information. Active parental consent was obtained prior to their children’s participation. Active consent was also obtained from adolescents of age, while their underage peers provided their assent to participate in the project. Adolescents completed seven assessments in January/February 2022 (T1), April/May 2022 (T2), September/October 2022 (T3), January/February 2023 (T4), April/May 2023 (T5), September/October 2023 (T6), and January/February 2024 (T7). Data were collected using repeated measurement windows each year (early year, spring, and autumn), resulting in a consistent pacing across waves. At each wave, researchers visited participants in schools during class hours and assisted them during the online questionnaire completion. Adolescents were required to create a personal code to ensure confidentiality and pair their answers over time. They were informed that participation was voluntary and that they could withdraw their consent at any time.

### Measures

Scale means, standard deviation, and reliability are reported in Table S3 of the Supplemental Materials. All scales displayed good reliability (αs ≥ 87).

#### Participants’ demographics

Information on participants’ demographics was collected at the first assessment. Adolescents provided information about their sex (0 = *male*, 1 = *female*), age (exact years at T1 based on adolescents’ date of birth), and their parents’ educational level (0 = *low*; 1 = *medium*; 2 = *high*). Additionally, they reported their own and their mother’s and father’s country of birth. Based on this information, adolescents were categorized as having either an ethnic majority Italian (0; i.e., those born in Italy with both parents born in Italy) or an ethnic minority (1; i.e., those born or living in Italy with at least one parent born outside Italy) background. All demographic variables were included as covariates in the sensitivity analyses.

#### Structural opportunity for intergroup contact in the classroom

A variable indicating the prevalence of ethnic outgroup members in each participating classroom was created through various steps. First, archive information on the prevalence of ethnic majority Italian and ethnic minority students in each of the participating classrooms was obtained from school principals at T1 or, when not available (for two schools), was estimated based only on students participating in the research project. Then, this information was combined with adolescents’ ethnic background to create the structural opportunity score. For ethnic majority Italian adolescents, the structural opportunity score corresponds to the percentage of ethnic minority students in the classroom. Conversely, for ethnic minority adolescents, the structural opportunity score reflects the prevalence of ethnic majority Italian students in the classroom.

#### Types of best friendships

At each wave, participants provided information about their best friend’s ethnic background by answering the question “Is your best friend Italian or from an ethnic minority background?”. The best friendship was not restricted to a specific context (i.e., adolescents’ best friend could be a classmate or schoolmate or not). First, this answer was paired with adolescents’ own ethnic background resulting in a time-specific variable distinguishing adolescents with an intragroup best friend (0; i.e., Italian adolescents with an Italian best friend, and ethnic minority adolescents with an ethnic minority best friend[ethnic minority based on shared ethnic minority status rather than specific ethnic origin]) and those with an intergroup best friend (1; i.e., Italian adolescents with an ethnic minority best friend and ethnic minority adolescents with an Italian best friend). Next, the type of best friendship was compared over time to create a nominal variable. Participants who indicated the same type of best friendship at each assessment were categorized as either 0 (*Stable intragroup best friendship*) or 1 (*Stable intergroup best friendship*). Those who reported a change in their best friendship type (e.g., from an intragroup best friendship at one time point to an intergroup best friendship at another time point) at least once were categorized as 2 (*Changing best friendship*).

#### Prejudice

Prejudice was assessed at each wave using the Feeling thermometer (Haddock et al., 1993; for the Italian version, see Bobba & Crocetti, 2022), which asks participants to rate how much they like different groups depending on their own ethnic background. Italian adolescents rated their liking of the most represented ethnic minority groups in the national context (i.e., Romanians, Albanians, Moroccans, Chinese, and Ukrainians; ISTAT, 2020) and foreign people in general (six items). Conversely, ethnic minority adolescents rated their liking of the group of Italians (one item). All participants answered on a scale from 0° (*not at all*) to 100° (*very much*). To simplify the presentation of results and avoid inflated variances, the scale was reversed (with higher scores indicating higher intergroup prejudice) and rescaled by dividing participants’ scores by 10.

#### Attitudes toward integration

Attitudes toward integration were assessed using the Attitudes Toward Migrant Integration Policies scale (AMIP; Maratia et al., 2024). It includes eight items based on the Migrant Integration Policy Index (MIPEX). Participants received this prompt: “You will be presented with several policies for the integration of people with a migrant background. Please, rate how important it is that Italian national programs support policies to foster…” followed by one item for each policy area, as for example “…family reunion (e.g., accommodation, residence period)”. For each item, adolescents rated their response on a Likert scale from 1 (*Not at all important*) to 5 (*Absolutely important*).

### Strategy of Analysis

Sample descriptives and attrition checks, and analyses for the first aim of the study were conducted in IBM SPSS Version 29 for Windows. The remaining analyses were conducted in M*plus* 8.10 (Muthén & Muthén, 2017), using the Maximum Likelihood Robust (MLR) estimator (Satorra & Bentler, 2001) and the *Type=Complex* function, which provides robust standard errors accounting for the nested structure of the data (i.e., students nested in classrooms). The dataset, analysis codes, and outputs of the current study can be retrieved from: 10.17605/OSF.IO/EMB6D. Regarding the first aim, the chi-square test was conducted to examine patterns of best friendship type among ethnic majority and minority youth. Standardized residuals were inspected to establish which observed values differed significantly from the expected ones.

To address the second aim (i.e., examining similarities and differences in developmental patterns of intergroup outcomes based on best friendship type), several steps were followed. First, the measurement invariance of prejudice and attitudes toward integration was assessed. Details about the procedure are reported in the Supplemental Materials. Second, Latent Growth Curve (LGC) models were tested in the full sample of participants separately for prejudice and attitudes toward integration to estimate the mean level (i.e., intercept) and rate of change (i.e., slope) of intergroup outcomes, as well as the variability in those parameters. To identify the model that best represents the development of constructs, increasingly complex LGC models were tested and compared against each other. First, an intercept-only model (M1), which assumes full stability of the psychological construct examined, is tested as a baseline model. Next, a linear slope model (M2), which assumes that the target variable changes linearly and constantly over time (i.e., factor loadings are fixed, starting from 0 and increasing by one unit every time point), is tested and its fit is compared against the previous model (M1). Then, a free-change model (M3), which assumes non-linear change over time (i.e., only two factor loadings are fixed for model identification, while the others are freely estimated), is tested and its fit compared against M2. Last, a quadratic slope model (M4), which implies constantly linear and quadratic change over time (i.e., all factor loadings are fixed; those of the linear slope start from 0 and increase of one unit at each time point, while those of the quadratic slope start from 0 and increase of a squared unit at each wave), is tested and compared against both M2 and M3. If both M3 and M4 provide good fit, the free-change model is retained for parsimony. The fit of each model was evaluated based on a combination of the following indices (Byrne, 2012): the Comparative Fit Index (CFI) with values higher than 0.90 and 0.95 indicative of acceptable and very good fit, respectively; the Root Mean Square Error of Approximation (RMSEA) and the Standardized Root Mean Residual (SRMR) with values below 0.08 and 0.05 indicative of an acceptable and very good fit, respectively. Additionally, the RMSEA’s 90% confidence interval’s upper bound lower than 0.10 indicates an acceptable model fit (Chen et al., 2008). For model comparison, two models are considered to be different if at least two of the following criteria were met: a Δχ_SB_^2^ significant at *p* < 0.05 (Satorra & Bentler, 2001), ΔCFI ≥ −0.010, and ΔRMSEA ≥ 0.015 (Chen, 2007). The best fitting and most parsimonious model was retained. This final model was tested again in a multigroup format to assess whether growth parameters (i.e., intercept and slope(s)) significantly differ between adolescents with an intragroup, intergroup, or changing best friendship type, while controlling for adolescents’ structural opportunity for contact in the classrooms by correlating this variable with the growth parameters. Multigroup modelling is an appropriate analytical choice when moderation effects are tested on multiple parameters (Memon et al., 2019). To this end, the growth parameters and their correlations were freely estimated for each best friendship group, and pair-wise differences across groups were inspected using the Wald test statistic. Specifically, a Wald test significant at *p* < 0.050 would indicate that adolescents in two types of best friendship groups significantly differ on a given parameter.

Last, multiple sensitivity checks were conducted. First, the main multigroup models were tested again, including participants’ ethnic background, sex, age, and parents’ educational level as covariates to examine whether differences across types of best friendship groups were maintained when accounting for the effect of demographics. Second, the main multigroup models were replicated by using a fine-grained categorization of types of best friendships to examine whether additional differences in developmental trajectories of prejudice and attitudes towards integration would emerge. The two sets of sensitivity analyses are further detailed in the Supplemental Materials.

## Results

### Preliminary Analyses

Correlations are reported in Table S4 of the Supplemental Materials. Regarding measurement invariance, full (for prejudice) and partial (for attitudes toward integration) scalar invariance were reached (Table S5). Thus, it was possible to proceed with the main analyses.

### Types of Best Friendships Over Time

The first goal of the study was to examine similarities and differences in the types of best friendships among ethnic majority (i.e., Italian) and ethnic minority adolescents. Results (Table 1) highlighted a significant difference in types of best friendships across ethnic groups (χ^2^(df) = 536.294(2), *p* < 0.001, φ = 0.661). While ethnic majority adolescents mostly reported stable intragroup best friendships, ethnic minority youth displayed a higher prevalence of either stable intergroup or changing best friendships.

**Table 1:** Types of best friendships and adolescents’ ethnic background

Types of best friendships	Ethnic majority adolescents	Ethnic minority adolescents
Stable intragroup (%)	82.23 (+)	14.49 (−)
Stable intergroup (%)	2.47 (−)	51.87 (+)
Changing best friend (%)	15.30 (−)	33.64 (+)

All observed values are significantly different from expected values based on the Chi-square test standardized residuals: (+) indicates that the observed value is higher than expected; (−) indicates that the observed value is lower than expected

### Implications of Types of Best Friendships

The second goal of the present study was to examine whether the types of best friendships throughout adolescence would moderate the development of prejudice and attitudes toward integration. Results of Latent Growth Curve Models on the total sample (Table 2) indicated that the quadratic slope and the free-change models were the best-fitting and most parsimonious solutions to represent change in prejudice and attitudes toward integration, respectively. Thus, they were used in subsequent multigroup analyses, controlling for structural opportunity for contact in the classroom.

**Table 2:** Latent Growth Curve models: Model fit and model comparison

	Model fit					Model comparison			
Models	χ_SB_^2^	df	CFI	SRMR	RMSEA [90% CI]	Models	Δχ_SB_^2^	ΔCFI	ΔRMSEA
**Prejudice**									
Intercept-only (M1)	214.682	26	0.915	0.071	0.077 [0.068, 0.087]				
Linear slope (M2)	128.132	23	0.953	0.049	0.061 [0.051, 0.072]	M1 – M2	78.031 (3)^***^	−0.038	0.016
Free-change (M3)	This model did not converge								
**Quadratic slope (M4)**	**73.912**	**19**	**0.975**	**0.028**	**0.049 [0.037, 0.061]**	**M2 – M4**	48.880 (4)^***^	**−0.022**	**0.012**
**Attitudes towards integration**									
Intercept-only (M1)	247.358	26	0.838	0.219	0.083 [0.074, 0.093]				
Linear slope (M2)	50.315	23	0.980	0.063	0.031 [0.019, 0.043]	M1 – M2	138.357 (3)^***^	−0.142	0.052
**Free-change (M3)**	**23.849**	**18**	**0.996**	**0.028**	**0.016 [0.000, 0.032]**	**M2 – M3**	25.058 (5)^***^	**−0.016**	**0.015**
Quadratic slope (M4)	26.965	19	0.994	0.054	0.019 [0.000, 0.033]	M2 – M4	23.378 (4)^***^	−0.014	0.012
						M3 – M4	2.616 (1)	−0.002	0.003

*M*, model*; χ*_*SB*_^*2*^, Satorra-Bentler scaled chi-square; *df*, degree of freedom; *CFI*, Comparative Fit Index; *SRMR*, Standardized Root Mean Square Residual; *RMSEA*, Root Mean Square Error of Approximation; *CI*, confidence interval; *Δ*, change in the parameter

^***^p < 0.001

#### Types of best friendships and prejudice

**Fig. 1:**
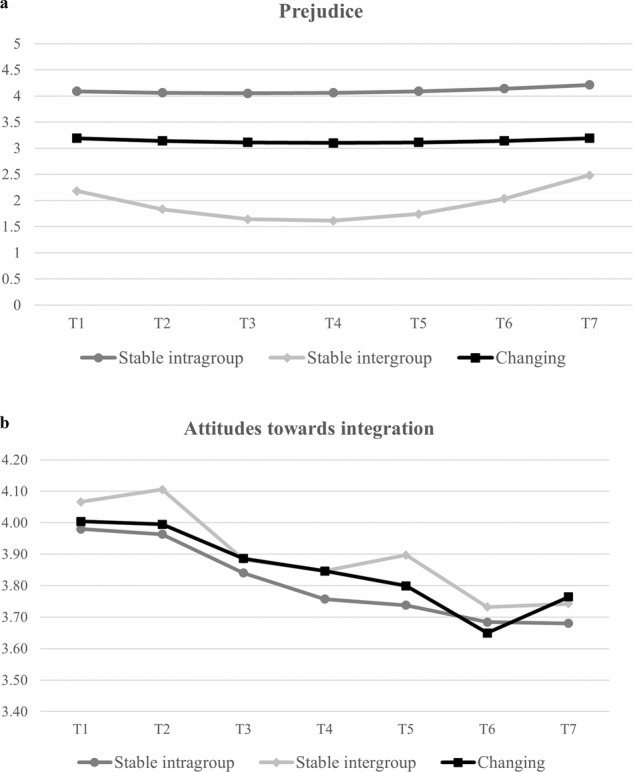
Implications of the types of best friendships for intergroup outcomes. *Note*. The assessment scales in the figures have been adjusted to facilitate the interpretation of findings. Prejudice was assessed on a scale from 0 to 10, while attitudes toward integration were measured on a scale from 1 to 5

The multigroup latent growth curve model of prejudice displayed an excellent fit: χ^2^(df) = 136.316(69), *p* < 0.001; CFI = 0.972; RMSEA [90% C.I.] = 0.049 [0.037, 0.061]; SRMR = 0.049. Results (see Table 3 and Fig. 1a) highlight several differences, mainly in growth parameters, across the three groups of adolescents. Regarding initial levels of prejudice, youth with a stable intragroup best friendship reported the highest scores compared to those with a changing (Wald = 16.54, *p* < 0.001) and those with a stable intergroup (Wald = 34.21, *p* < 0.001) best friendship type. Furthermore, the latter reported significantly lower initial levels of prejudice compared to their peers with a changing best friendship (Wald = 9.34, *p* = 0.002). Regarding patterns of change, no differences emerged in the linear slope. However, adolescents with a stable intergroup best friendship type displayed a dip in prejudice scores due to a significant quadratic slope, which differed from that of adolescents with a stable intragroup (Wald = 4.24, *p* = 0.039) and those with changing best friendship type (Wald = 3.97, *p* = 0.043). Additionally, a small significant difference emerged in the association between structural opportunity for intergroup contact in classroom and intercept scores between adolescents with a changing best friendship type and those with a stable intragroup best friendship type (Wald = 6.59, *p* = 0.010). Specifically, for the former but not for the latter, having more opportunity for contact with the outgroup in class was associated with significantly lower levels of prejudice at the beginning of the study.

**Table 3:** Implications of types of best friendships for intergroup outcomes

	Total sample	Types of best friendships		
		Stable intragroup	Stable intergroup	Changing best friend
**Prejudice**				
**Growth parameters*M*(*σ***^***2***^**)**				
Intercept	3.72^***^ (5.31^***^)	4.09_a_^***^ (5.26^***^)	2.18_c_^***^ (3.04^*^)	3.19_b_^***^ (4.87^***^)
Linear slope	−0.08 (0.74^***^)	−0.04 (0.68^***^)	−0.43^*^ (0.72)	−0.06 (0.87^**^)
Quadratic slope	0.02^*^ (0.02^***^)	0.01_b_ (0.02^***^)	0.08_a_^**^ (0.02)	0.01_b_ (0.02^*^)
**Standardized correlations**				
Opportunity **↔** Intercept		−0.02_b_	−0.27_a,b_^*^	−0.32_a_^**^
Opportunity **↔** Linear slope		−0.05	0.08	−0.04
Opportunity **↔** Quadratic slope		0.05	−0.10	0.04
**Attitudes toward Integration**				
**Growth parameters*M*(*σ***^***2***^**)**				
Intercept	3.99^***^ (0.25^***^)	3.98^***^ (0.26^***^)	4.07^***^ (0.18^***^)	4.00^***^ (0.26^***^)
Slope	−0.05^***^ (0.00^***^)	−0.05^***^ (0.00^***^)	−0.05^***^ (0.00)	−0.04^***^ (0.00)
**Standardized correlations**				
Opportunity **↔** Intercept		−0.04	0.13	−0.06
Opportunity **↔ **Slope		−0.08	−0.20	0.04

Subscript letters within the same line indicate significant differences between adolescents in the three groups either in means or correlation coefficients. Subscript letters in parentheses indicate that the Wald test was only marginally significant.

^*^p < 0.05; ^**^p < 0.01; ^***^p < 0.001

#### Types of best friendships and attitudes toward integration

The multigroup latent growth curve model of attitudes toward integration displayed a very good fit: χ^2^(df) = 78.201(69), *p* = 0.210; CFI = 0.995; RMSEA = 0.018 [0.000, 0.035]; SRMR = 0.060. Results are reported in Table 3 and displayed in Fig. 1b. As can be inferred, all adolescents showed positive attitudes towards integration which decreased over time. This developmental trajectory was identical for adolescents with stable intragroup, stable intergroup, and changing best friendship types. Furthermore, opportunity for contact with the outgroup in the classroom was not significantly associated with initial levels and rates of change in attitudes towards integration for any of the types of best friendships groups.

### Sensitivity Analyses

Results (see Table S6) of multigroup models with covariates largely replicated the main findings for both prejudice and attitudes towards integration. Only two notable differences emerged regarding the effect of structural opportunity for intergroup contact in the classroom. First, its effect on the initial levels of prejudice became not significant for adolescents in the stable intergroup and in the changing best friendship groups. Second, this factor was linked to steeper decreases in attitudes towards integration only among adolescents with a stable intergroup best friendship.

Results (see Table S7, Figure S1) of the multigroup model of prejudice with an alternative grouping of best friendship trajectories largely replicated the main findings and, interestingly, no differences emerged across the three groups that were derived from the “changing best friendship” type (lost intergroup best friendship, gained intergroup best friendship, and multiple shifts). Thus, the three types of best friendships used in the main analyses provide a parsimonious and meaningful representation of heterogeneity in adolescents’ experiences.

## Discussion

Establishing contact with peers from diverse ethnic backgrounds is widely recognized as a key mechanism through which adolescents form positive intergroup attitudes (Allport, 1954; Davies et al., 2011), successfully navigating the complexity of current multicultural societies. However, little is known about patterns of stability in intragroup and intergroup best friendships throughout adolescence, and how they contribute to the development of intergroup attitudes. To address this gap, the present study examined intragroup and intergroup friendship patterns of ethnic majority and minority adolescents longitudinally and the extent to which stability of these friendships is intertwined with changes in prejudice and attitudes toward integration. Overall, the findings revealed meaningful differences between ethnic majority and minority adolescents in the stability of their friendship ties, which, in turn, shaped how their intergroup attitudes developed over time.

### Stability of Intragroup and Intergroup Friendships: Different Social Processes for Ethnic Majority and Minority Youth

The first aim of this study was to understand whether intragroup and intergroup best friendships of ethnic majority and minority adolescents remained stable over time. The findings revealed significant differences in the prevalence and stability of best friendships across ethnic groups. Thus, different social processes might sustain the formation and maintenance of friendships depending on adolescents’ backgrounds.

In partial support of Hypothesis 1, stable intragroup best friendships were found to be more prevalent among ethnic majority adolescents. This finding aligns with the homophily principle (McPherson et al., 2001) and extends its implications to the maintenance of friendship ties over time. In other words, ethnic majority youth not only prefer best friends who are similar to them in terms of ethnic background but also maintain such friendships more consistently. Furthermore, this finding is consistent with prior empirical research indicating that intragroup friendships during adolescence are generally more prevalent (Kelleghan et al., 2019) and stable (e.g., Oczlon et al., 2023).

Conversely, ethnic minority adolescents were more likely to form and maintain intergroup best friendships. This result contrasts with some prior studies that highlight the lower quality (McGill et al., 2012) and reduced stability (Jugert et al., 2013) of intergroup friendships. However, this pattern can be explained in light of the unique characteristics of the Italian socio-cultural context. First, in the Italian educational context, which replicates a larger societal pattern, most students have an ethnic majority background (Ministero della Pubblica Istruzione, 2024). Thus, in line with the principle of propinquity (Mouw & Entwisle, 2006), ethnic minority adolescents tend to form best friendship ties with those who are physically present and numerically prevalent in their contexts of development. Second, ethnic minority youth in Italy are predominantly second-generation immigrants, who are often well-integrated into the host society linguistically and culturally (Karataş et al., 2023). This strong social embeddedness might support the formation of intergroup best friendships characterized by more intimate and closer bonds, which are conducive to longer-term stability (Brown, 2004).

Moreover, this study also found that the types of best friendships of ethnic minority adolescents exhibited a certain degree of instability. Although over half maintained stable best friendships (either intragroup or intergroup), approximately one-third experienced changes in the type of friendship over the study period. The pattern may partly reflect heterogeneity within the minority group, as a proportion of participants were first-generation immigrants (approximately 26% of the minority subsample). For these adolescents, adapting to the host society often involves balancing integration into the mainstream culture with the maintenance of heritage cultural traditions, which may entail additional identity exploration, questioning, and reconstruction (Crocetti et al., 2011, 2024). During this development process, adolescents are likely to adjust their social relationships in response to evolving developmental needs (Benner, 2011). Such ongoing interpersonal exploration and relational adjustment have been shown to increase the risk of friendship dissolution (Jugert et al., 2013), thereby contributing to greater relational fluidity. In addition, previous research indicates that out-of-school contact is a crucial condition for the maintenance and durability of intergroup friendships (Lessard et al., 2019). However, for some ethnic minority adolescents who have not yet fully integrated into the host society, opportunities to sustain interactions beyond the school context may be limited, which may further undermine the stability of their friendships.

### Best Friendship and the Development of Intergroup Attitudes in Multicultural Contexts

The second goal of the present study was to investigate whether the types (intragroup vs. intergroup) and stability of best friendships influence the development of adolescents’ prejudice and attitudes toward integration. The findings only partially supported the expectations. Specifically, they highlight that patterns of best friendship might be more relevant in shaping negative (i.e., prejudice) compared to positive (i.e., attitudes toward integration) intergroup attitudes.

#### Types of best friendships shape adolescents’ prejudice

Consistent with Hypothesis 2a, adolescents were found to display a unique developmental trajectory of intergroup prejudice depending on the type and stability of their best friendship. Specifically, adolescents who maintained stable intergroup best friendships exhibited the lowest initial levels of prejudice, which declined over time. This finding aligns with previous research, suggesting that sustained and intimate intergroup friendships constitute a powerful resource for reducing prejudice (Rastogi & Juvonen, 2019). Importantly, this decline was nonlinear, with a steeper reduction occurring during the initial stages and a more gradual increase thereafter. This pattern suggests that early intergroup interactions may be especially effective in challenging stereotypes and promoting cognitive reevaluation (Davies et al., 2011; Vezzali et al., 2017). As intergroup contact becomes increasingly normalized, its incremental benefits for prejudice reduction may weaken over time, leading to diminishing reductions in prejudice (Raabe & Beelmann, 2011).

In contrast, adolescents with stable intragroup best friendships showed the highest initial levels of intergroup prejudice, which remained stable over time. Forming friendships with similar peers might strengthen processes of ingroup identification (Graham et al., 2014), which in turn can contribute to the development and maintenance of negative intergroup attitudes (Bobba et al., 2024b). Furthermore, youth who engage in stable intragroup friendships might have less knowledge of and familiarity with outgroup members, key factors that contribute to prejudicial attitudes and views of others (Pettigrew & Tropp, 2008). Notably, this group of adolescents still displayed levels of prejudice that were below the mid-point of the scale, highlighting the generally positive views of others.

Adolescents whose best friendship type changed exhibited levels of prejudice that consistently fell between those of the other two groups throughout the study period. The shift in friendship type indicates that adolescents’ best friends alternated between ingroup and outgroup peers. This fluidity provides adolescents with diverse intergroup contact opportunities and perspectives, which may lead to lower prejudice (Killen et al., 2021; Pettigrew & Tropp, 2006). However, the lack of continuity in intergroup contact may hinder the accumulation of familiarity and emotional security, thereby limiting the potential for prejudice reduction (Crystal et al., 2008; Stark et al., 2013). Consequently, their intergroup prejudice remains moderate and relatively stable, neither decreasing substantially nor reaching extreme levels.

It is worth noting that structural opportunity for intergroup contact in the classroom did not affect all adolescents uniformly. Instead, their efficacy appears contingent upon the stability and type of adolescents’ best friendships (Smith et al., 2016). For those with dynamic friendship patterns (changing best friend) and intergroup friendships, relational networks are more flexible and open, which makes it more likely that classroom contact opportunities translate into actual intergroup interactions, resulting in lower initial levels of prejudice (Titzmann et al., 2015). In contrast, stable intragroup best friendships may reinforce homogeneous network structures and strengthen group boundaries (Graham et al., 2014; McPherson et al., 2001), thereby limiting the influence of structural contact opportunities on prejudicial attitudes (Davies et al., 2011). Importantly, these effects were observed primarily during the early stages of prejudice formation, suggesting that while structural opportunities shape initial attitudes, the long-term development of prejudice depends more on sustained, ongoing friendship interactions.

#### Types of best friendships are not related to the development of attitudes toward integration

Contrary to Hypothesis 2b, attitudes toward integration showed a similar developmental trajectory regardless of adolescents’ type of best friendship. Specifically, all youth displayed high initial levels of positive attitudes, which significantly, albeit slightly, decreased over time. In addition, structural opportunity for intergroup contact in the classroom did not exert a significant effect on attitudes toward integration. This finding suggests that the development of attitudes toward integration may be influenced by other mechanisms at work in multicultural societies.

Specifically, the high baseline of attitudes toward integration likely reflects Italian society’s longstanding emphasis on inclusive values, particularly through the extensive implementation of inclusive education in schools (European Agency for Special Needs and Inclusive Education, 2021). This inclusion environment enables both majority and minority adolescents to internalize positive attitudes toward inclusion early in their value development (Albarello et al., 2023). Notably, adolescents’ scores on attitudes toward integration were close to 4 on a 5-point Likert scale across all measurement points, and differences between groups were minimal. Statistically, this pattern may indicate some restriction of range at the upper end of the construct, potentially reducing the model’s sensitivity in detecting subtle differences among friendship types. Consequently, nonsignificant effects should be interpreted with caution, also considering possible ceiling effects (Wang et al., 2008).

Intriguingly, adolescents’ attitudes toward integration declined slightly over time. This decline may be associated with normative developmental changes in adolescents’ cognitive maturation and increasing political awareness. As adolescents mature, their capacity to engage in more nuanced evaluation and critical reflection on sociopolitical issues tends to increase (Flanagan, 2013; Rekker et al., 2015). This developmental progression may prompt adolescents to assess existing integration policies in a more differentiated and cautious manner, even if the mechanisms underlying their perceptions of policy limitations differ (Sidler et al., 2022). Overall, the slight decline in integration attitudes is more likely to reflect the maturation of adolescents’ sociopolitical reasoning and the influence of broader societal factors, rather than differences in types of friendships or ethnic background.

However, neither types of best friendships nor classroom structure had a significant influence on the developmental trajectories of attitudes toward integration. Conceptually, attitudes toward integration reflect adolescents’ support for policies promoting the integration of migrants, thus tapping into youth socio-political views (Maratia et al., 2024). The development of these orientations occurs within and is influenced by experiences in key proximal developmental contexts, such as the family (Bronfenbrenner, 1992, 2005). Previous research has highlighted intergenerational continuity in political preferences and voting (e.g., Iyengar et al., 2018) and transmission of attitudes toward integration from parents to their adolescent offspring (Maratia & Crocetti, 2024). Thus, the best friendship type might be less relevant in directly shaping youth’s endorsement of integration policies. In addition, it is worth noting that the majority of ethnic minority adolescents in the present sample were second-generation immigrants (73.8%), a group that is generally more integrated into the host society and tends to hold socio-political orientations closer to those of majority peers (Barrera-Rodríguez et al., 2025; Berry, 1997). This sociopolitical convergence may reduce the potential impact of peer and classroom environment differences on the development of attitudes toward integration.

### Limitations and Suggestions for Future Research

The present findings should be interpreted considering some limitations. First, the sample was drawn from the Emilia-Romagna region in northeastern Italy, where the proportion of ethnic minority students in school is relatively high compared to other regions (Ministero della Pubblica Istruzione, 2024). This regional characteristic may limit the generalizability of the findings. Future research should extend the geographical scope and replicate the study across countries and regions with different levels of ethnic and cultural diversity.

Second, the classification of friendship types in this study was relatively limited, distinguishing only between stable intragroup, stable intergroup, and changing best friendships. For instance, for ethnic minority adolescents it was not possible to differentiate between those who have as a best friend an adolescent from the same ethnic group (e.g., friendship between two Chinese adolescents) or from a different one (e.g., friendship between a Chinese adolescent and an Albanian adolescent). Inter-minority best friendship ties may carry different developmental implications (Rastogi & Juvonen, 2019) that could not be tackled. Furthermore, adolescents in the changing group showed different trajectories of best friendship type, which might have unique implications for the development of intergroup attitudes and behaviors. Although no differences emerged in the sensitivity analyses with prejudice, further research with larger samples of adolescents is needed to corroborate this evidence. Additionally, the current study did not rely on peer nomination techniques and it was thus not possible to determine whether the same best friend was maintained over time (Poulin & Dishion, 2008). Rather, the focus of this research was on the stability of intragroup and intergroup best friendship ties. Future research would benefit from employing peer nomination and examining the quality and stability of friendships from a dyadic perspective.

Third, the current study highlighted differences in the prevalence of stable intragroup, stable intergroup, and changing best friendship types among adolescents with different ethnic backgrounds. However, due to the limited number of participants, especially those with an ethnic minority background, it was not possible to examine whether different types of best friendships had unique implications for the development of prejudice and attitudes towards integration among adolescents with ethnic majority and ethnic minority backgrounds. Future studies should strive for larger and more balanced samples of diverse adolescents to provide a more fine-grained understanding of the implications of best friendship ties in multicultural contexts.

Fourth, this study did not account for a range of individual and contextual factors that may affect the formation and stability of adolescent intragroup and intergroup friendships. These include outside-of-school interactions (Lessard et al., 2019), parents’ influence (Maratia & Crocetti, 2024), as well as engagement with digital media (Turetsky & Shelton, 2024). Further research is needed to explore the determinants of stable friendship patterns during adolescence. Understanding these factors is crucial for supporting the development of lasting friendships and fostering inclusive social environments.

Finally, the current study relied on a single-item measure to assess ethnic minority adolescents’ prejudice against the ethnic majority group. In the Italian context, nationality and citizenship are tied to ethnic and cultural heritage (Reijerse et al., 2013) and, as such, adolescents were expected to interpret the label “Italian” with reference to the ethnic majority population. However, at least some of the participants might have also interpreted this term differently, which may have introduced conceptual ambiguity and attenuated observed associations.

## Conclusion

In increasingly multicultural societies, adolescents are gaining more opportunities to form intergroup friendships. This study examined the stability of adolescents’ intragroup and intergroup best friendships and their implications for the development of negative (i.e., prejudice) and positive (i.e., attitudes toward integration) intergroup attitudes. Ethnic majority adolescents were more likely to engage in stable intragroup best friendships, whereas their ethnic minority peers were either more likely involved in stable intergroup best friendships or changed their type of friendship over time. Adolescents displayed unique developmental trajectories of prejudice depending on the type and stability of the best friendship they engaged with, while this relational characteristic did not shape the development of attitudes toward integration. These findings underscore the importance of stable intergroup friendships in preventing the development of negative attitudes in multicultural societies.

## Supplementary information

Supplemental materials

## Data Availability

The datasets analyzed during the current study are available in the Open Science Framework repository:https://doi.org/10.17605/OSF.IO/EMB6D.
