# Supplementary material for: Intergroup, Intragroup, and Changing Best Friendships: Implications for Prejudice and Attitudes Toward Integration in Ethnic Majority and Minority Adolescents
Source: J Youth Adolesc. 2026 Apr 15;55(7):1789–803. doi: 10.1007/s10964-026-02354-5 (PMC13328150; doi:10.1007/s10964-026-02354-5)
Supplement: Supplementary file 1 — Supplemental materials [file 10964_2026_2354_MOESM1_ESM.docx]

**SUPPLEMENTAL MATERIALS**

**Intergroup, Intragroup, and Changing Best Friendships: Implications for Prejudice and Attitudes Toward Integration in Ethnic Majority and Minority Adolescents**

| Group comparisons and ethnic minority participants’ demographics | 2 |
| --- | --- |
| Sample attrition analyses | 3 |
| Table S1. Results of Chi-square analyses | 4 |
| Table S2. Results of univariate ANOVAs | 5 |
| Table S3. Scale descriptives and reliability | 6 |
| Table S4. Correlations among study variables | 7 |
| Longitudinal measurement invariance | 8 |
| Table S5. Measurement invariance of study variables: Model fit and model comparison | 9 |
| Sensitivity analyses: Models with covariates | 10 |
| Table S6. Results of models with covariates | 12 |
| Sensitivity analyses: Alternative grouping of best friendship types | 13 |
| Table S7. Results of models with alternative groups | 16 |
| Figure S1. Development of prejudice across five types of best friendships | 17 |

**Group comparisons and ethnic minority participants’ demographics**

The current study involved 1,013 adolescents with an ethnic majority Italian background and 214 adolescents with an ethnic minority background. Chi-square analyses and t-tests were conducted to examine possible differences among the two groups of participants in terms of demographic characteristics. Regarding sex differences, the subsample of Italian adolescents comprised slightly fewer female participants compared to the ethnic minority subsample (χ^2^(1) = 4.26, *p* = .039). For what concerns age, adolescents with an Italian background were slightly younger compared to their ethnic minority peers (t(1.14) = -2.83, *p* = .005). Furthermore, regarding parental education, significant differences emerged in mothers’ (χ^2^(2) = 26.07, *p* < .001) but not in fathers’ educational levels (χ^2^(2) = 5.35, *p* = .069) across the two groups. Specifically, a higher proportion of mothers of ethnic minority adolescents (31.11%) reported low levels of education (i.e., up to middle school diploma) compared to mothers of Italian youth (15.31%).

Within the subsample of ethnic minority adolescents, a quarter (26.20%) were first-generation and almost three quarters (73.80%) were second-generation youth who were born in Italy. Regarding first-generation ethnic minority adolescents, most (33.33%) were born in Eastern European countries (e.g., Albania, Romania, Moldova), followed by those (33.33%) from Asian (e.g., India, Pakistan, China) and by those (22.90%) from African (e.g., Nigeria, Ghana, Egypt) countries. The remaining were born in Central European (6.24%; e.g., Germany), North (2.10%; e.g., USA), or South (2.10%; e.g., Brazil) American countries. Similarly, parents of most second-generation students came from Eastern European (e.g., Albania, Romania; 41.81% of mothers and 35.10% of fathers) or African (e.g., Morocco, Nigeria; 23.80% of mothers and 34.04% of fathers) countries, followed by those from Asian (e.g., China, India; 11.50% of mothers and 14.88% of fathers) and South American (e.g., Argentina, Brazil; 10.66% of mothers and 7.43% of fathers) ones. The remaining few came from Central Europe (e.g., Germany; 9.84% of mothers and 8.55% of fathers) or North America (e.g., USA; 1.64% of mothers). Overall, the sample characteristics reflect the socio-demographics of the national (ISTAT, 2022) and local (i.e., Emilia-Romagna region; Regione Emilia-Romagna, 2022) contexts. Specifically, the most represented ethnic minority groups include those from Eastern Europe (e.g., Albania, Romania, and Ukraine), Asia (e.g., China, Bangladesh, India), and Africa (e.g., Morocco).

**Sample attrition analyses**

The current study included adolescents’ assessments at seven time points between January/February 2022 and January/February 2024. Out of the participating adolescents, 15.73% completed only two assessments, 10.19% completed three, 11.98% completed four, 10.84% participated in five assessments, 20.70% in six, and 30.56% completed questionnaires at all time points. Sample attrition was further inspected by means of Chi-square tests and Univariate ANOVAs to identify possible differences in demographics and study variables among adolescents with varying participation rates.

A set of chi-square tests was conducted to examine differences in adolescents’ sex and ethnic background, in their parents’ educational level, and in the prevalence of different types of best friendship (i.e., stable intragroup, stable intergroup, and changing best friend). Results are displayed in Table S1. As can be inferred, adolescents with varying degrees of wave participation differed significantly on all demographic indicators as well as on their type of best friendship. However, effects were small in magnitude, with the most evident difference in youth’s school track. Particularly, adolescents attending vocational school tracks and those following a technical track were more likely to participate in a lower number of waves compared to those in academic-oriented tracks. Regarding types of best friendship, adolescents with stable intergroup best friendship were more likely to have participated only twice and less likely to have completed all study assessments.

Further, univariate ANOVAs were conducted to test whether adolescents with varying degrees of participation differed in their mean age (at T1) and in their levels of prejudice and attitudes towards integration (aggregated across study occasions). Results are displayed in Table S2. As can be inferred, adolescents with varying participation rates significantly differ in their age at T1 and scores of attitudes towards integration and prejudice. Specifically, adolescent who participated only twice were slightly older than those who completed six assessments, while adolescents who completed either six or seven waves showed on average slightly lower prejudice and more positive attitudes towards integration. However, the magnitude of these effects was very small.

**Table S1**

Results of Chi-square analyses

| Adolescent participation in… | | | | | | | | | | |
| --- | --- | --- | --- | --- | --- | --- | --- | --- | --- | --- |
|  | 2 waves | 3 waves | 4 waves | 5 waves | 6 waves | 7 waves | χ^2^ | df | *p* | Cramer’s V |
| **Adolescent sex** |  |  |  |  |  |  | 17.223 | 5 | .004 | .119 |
| Male | 107 | **79 (+)** | 88 | 63 | 118 | 188 |  |  |  |  |
| Female | 82 | **44 (-)** | 59 | 70 | 136 | 187 |  |  |  |  |
| **Adolescent school type** |  |  |  |  |  |  | 136.369 | 10 | < .001 | .236 |
| Academic-oriented | **48 (-)** | **26 (-)** | **38 (-)** | 50 | **139 (+)** | **208 (+)** |  |  |  |  |
| Technical | **81 (-)** | **64 (+)** | **82(+)** | 45 | **81 (-)** | 129 |  |  |  |  |
| Vocational | **64 (+)** | **35 (+)** | 27 | **38 (+)** | **34 (-)** | **38 (-)** |  |  |  |  |
| **Adolescent background** |  |  |  |  |  |  | 19.662 | 5 | .001 | .127 |
| Italian descent | 144 | 98 | 118 | 108 | 214 | 331 |  |  |  |  |
| Immigrant descent | **49 (+)** | 27 | 29 | 25 | 40 | **44 (-)** |  |  |  |  |
| **Father’s educational level** |  |  |  |  |  |  | 23.918 | 10 | .008 | .104 |
| Up to middle school diploma | 40 | 35 | 28 | **43 (+)** | 60 | 91 |  |  |  |  |
| High school diploma | 95 | 49 | 56 | 54 | 120 | 167 |  |  |  |  |
| University degree or higher | 33 | 19 | 20 | **19 (-)** | 59 | **111 (+)** |  |  |  |  |
| **Mother’s educational level** |  |  |  |  |  |  | 32.191 | 10 | < .001 | .121 |
| Up to middle school diploma | **46 (+)** | **29 (+)** | 22 | 23 | **29 (-)** | **48 (-)** |  |  |  |  |
| High school diploma | 78 | 46 | 48 | 57 | 131 | 181 |  |  |  |  |
| University degree or higher | 46 | 28 | 35 | 37 | 78 | 139 |  |  |  |  |
| **Types of best friendships** |  |  |  |  |  |  | 40.713 | 10 | < .001 | .129 |
| Stable intragroup | 129 | 83 | 106 | 87 | 182 | 277 |  |  |  |  |
| Stable intergroup | **42 (+)** | 15 | 18 | 13 | 25 | **23 (-)** |  |  |  |  |
| Changing best friend | **22 (-)** | 27 | 23 | 33 | 47 | 75 |  |  |  |  |

*Note.* Counts are reported in cells. Bold values indicate that the observed count is significantly different from the expected count based on standardized residuals: (+) indicates that the observed value is higher than expected, while (-) indicates that the observed value is lower than expected. Cramer’s V is reported as an effect size for Chi-square tests (values ≈ .10 indicate a small effect, ≈ .30 a medium effect, and ≈ .50 a large effect; Cohen, 1988).

**Table S2**

Results of univariate ANOVAs

| Adolescent participation in… | | | | | | | | | | |
| --- | --- | --- | --- | --- | --- | --- | --- | --- | --- | --- |
|  | 2 waves | 3 waves | 4 waves | 5 waves | 6 waves | 7 waves | *F* | df | *p* | η^2^ |
| **Adolescent age at T1** | 15.94 _b_  (1.31) | 15.74 _a, b_  (1.36) | 15.56 _a ,b_  (1.19) | 15.83  _a, b_  (1.21) | 15.52 _a_  (1.09) | 15.60 _a, b_  (1.15) | 3.585 | 5 | .003 | .015 |
| **Attitudes towards integration** | 3.75 _a, b_  (0.60) | 3.72 _a_  (0.59) | 3.70 _a_  (0.57) | 3.84 _a, b, c_  (0.64) | 3.90 _b, c_  (0.53) | 3.94 _c_ (0.55) | 6.960 | 5 | < .001 | .028 |
| **Prejudice** | 4.25  _a, b_  (2.51) | 3.71 _a, b_  (2.58) | 4.25 _b_  (2.65) | 3.53 _a, b_  (2.56) | 3.51 _a, b_  (2.40) | 3.49 _a_  (2.42) | 4.192 | 5 | < .001 | .017 |

*Note.* Means and standard deviations (in parenthesis) are reported in the cells. Within the same row, means with different subscripts differ significantly at *p* < .05 based on post-hoc comparisons, with letters in alphabetical order corresponding to increasingly higher mean scores. Attitudes towards integration was measured on a scale from 1 to 5, while prejudice was assessed on a scale from 0 to 10.

**Table S3**

Scale descriptives and reliability

|  | **Attitudes toward integration** | | | **Prejudice** | | |
| --- | --- | --- | --- | --- | --- | --- |
|  | *M* | *SD* | α | *M* | *SD* | α^1^ |
| T1 | 4.03 | 0.72 | .91 | 3.81 | 2.84 | .92 |
| T2 | 4.00 | 0.65 | .87 | 3.44 | 2.76 | .92 |
| T3 | 3.91 | 0.68 | .90 | 3.32 | 2.74 | .93 |
| T4 | 3.81 | 0.71 | .91 | 3.79 | 2.91 | .94 |
| T5 | 3.80 | 0.70 | .90 | 3.66 | 2.92 | .94 |
| T6 | 3.75 | 0.73 | .92 | 3.79 | 2.94 | .94 |
| T7 | 3.79 | 0.73 | .92 | 3.60 | 2.97 | .95 |

*Note.* Means and standard deviations were computed in M*plus* accounting for the nested structure of the data with *Type=Complex*. T= Time; M = Mean; SD = Standard deviation; α = Cronbach’s alpha. ^1^Cronbach’s alpha for the prejudice scale are calculated only for ethnic majority Italian respondents since the scale for ethnic minority youth includes only a single item.

**Table S4**

Standardized correlations among study variables

|  | 1. | 2. | 3. | 4. | 5. | 6. | 7. | 8. | 9. | 10. | 11. | 12. | 13. | 14. | 15. | 16. |
| --- | --- | --- | --- | --- | --- | --- | --- | --- | --- | --- | --- | --- | --- | --- | --- | --- |
| 1. Sex |  |  |  |  |  |  |  |  |  |  |  |  |  |  |  |  |
| 2. Age | .03 |  |  |  |  |  |  |  |  |  |  |  |  |  |  |  |
| 3. Ethnic background | .06 | .08^*^ |  |  |  |  |  |  |  |  |  |  |  |  |  |  |
| 4. Attitudes vs. integration T1 | .19^***^ | -.03 | .03 |  |  |  |  |  |  |  |  |  |  |  |  |  |
| 5. Attitudes vs. integration T2 | .17^***^ | .02 | .07^*^ | **.54^***^** |  |  |  |  |  |  |  |  |  |  |  |  |
| 6. Attitudes vs. integration T3 | .13^***^ | .03 | .04 | .46^***^ | **.55^***^** |  |  |  |  |  |  |  |  |  |  |  |
| 7. Attitudes vs. integration T4 | .19^***^ | .02 | .01 | .50^***^ | .53^***^ | **.65^***^** |  |  |  |  |  |  |  |  |  |  |
| 8. Attitudes vs. integration T5 | .21^***^ | .08 | .06 | .47^***^ | .54^***^ | .62^***^ | **.65^***^** |  |  |  |  |  |  |  |  |  |
| 9. Attitudes vs. integration T6 | .16^***^ | .07 | .00 | .47^***^ | .50^***^ | .58^***^ | .60^***^ | **.64^***^** |  |  |  |  |  |  |  |  |
| 10. Attitudes vs. integration T7 | .23^***^ | .08 | .03 | .48^***^ | .51^***^ | .58^***^ | .64^***^ | .67^***^ | **.72^***^** |  |  |  |  |  |  |  |
| 11. Prejudice T1 | -.17^***^ | -.02 | -.28^***^ | -.41^***^ | -.35^***^ | -.29^***^ | -.28^***^ | -.35^***^ | -.30^***^ | -.34^***^ |  |  |  |  |  |  |
| 12. Prejudice T2 | -.15^***^ | -.01 | -.25^***^ | -.34^***^ | -.43^***^ | -.38^***^ | -.34^***^ | -.35^***^ | -.30^***^ | -.33^***^ | **.63^***^** |  |  |  |  |  |
| 13. Prejudice T3 | -.16^***^ | -.02 | -.30^***^ | -.40^***^ | -.41^***^ | -.42^***^ | -.39^***^ | -.39^***^ | -.39^***^ | -.39^***^ | .66^***^ | **.72^***^** |  |  |  |  |
| 14. Prejudice T4 | -.16^***^ | -.06 | -.30^***^ | -.32^***^ | -.36^***^ | -.40^***^ | -.38^***^ | -.41^***^ | -.33^***^ | -.38^***^ | .59^***^ | .66^***^ | **.79^***^** |  |  |  |
| 15. Prejudice T5 | -.15^***^ | -.03 | -.29^***^ | -.35^***^ | -.39^***^ | -.35^***^ | -.39^***^ | -.41^***^ | -.36^***^ | -.39^***^ | .57^***^ | .69^***^ | .75^***^ | **.80^***^** |  |  |
| 16. Prejudice T6 | -.19^***^ | -.09^*^ | -.30^***^ | -.38^***^ | -.32^***^ | -.28^***^ | -.32^***^ | -.33^***^ | -.37^***^ | -.38^***^ | .57^***^ | .63^***^ | .68^***^ | .71^***^ | **.77^***^** |  |
| 17. Prejudice T7 | -.21^***^ | -.03 | -.26^***^ | -.37^***^ | -.33^***^ | -.35^***^ | -.32^***^ | -.36^***^ | -.36^***^ | -.41^***^ | .57^***^ | .61^***^ | .68^***^ | .67^***^ | .74^***^ | **.79^***^** |

*Note.* Correlations were computed in M*plus* accounting for the nested structure of the data with *Type=Complex*.

T = Time; Sex: 0 = male, 1 = female; Ethnic Background: 0 = ethnic majority, 1 = ethnic minority. Blue-bolded values indicate rank-order stability coefficients of attitudes toward integration. Black-bolded values indicate rank-order stability coefficients of prejudice.

^*^ *p* < .05; ^**^ *p* < .01; ^***^ *p* < .001.

**Longitudinal Measurement Invariance**

As a preliminary step, configural, metric, and scalar levels of measurement invariance were tested for prejudice and attitudes toward integration, separately. To this end, the configural models are first estimated as baseline models, and their fit was evaluated based on the following criteria: The Comparative Fit Index (CFI) with values higher than .90 and .95 indicate acceptable and very good fit, respectively. The Root Mean Square Error of Approximation (RMSEA) and the Standardized Root Mean Residual (SRMR) with values below .08 and .05 are indicative of an acceptable and very good fit, respectively (Byrne, 2012). Additionally, the RMSEA’s 90% confidence interval’s upper bound lower than .10 indicates an acceptable fit of the model (Chen et al., 2008). In order to establish metric (i.e., factor loadings constrained to be equal across times) and scalar (i.e., intercepts constrained to be equal across times) invariance, changes in fit indices from the configural to the metric model and from the metric to the scalar model were evaluated (e.g., Cheung & Rensvold, 2002). Specifically, if at least two out of the three following criteria were satisfied, this would be indicative of non-invariance: (a) Δχ_SB_^2^ significant at *p* < .05, (b) ΔCFI ≥ -.010, and (c) ΔRMSEA ≥ .015 (Chen, 2007; Satorra & Bentler, 2001). Results are displayed in Table S5. As can be inferred, prejudice displayed full scalar invariance while attitudes toward integration reached partial scalar invariance. Based on these results, we could proceed with the main analyses.

**Table S5**

Measurement invariance of study variables: Model fit indices and model comparisons

| Models | Model fit | | | | |  |  | Model comparisons | | |
| --- | --- | --- | --- | --- | --- | --- | --- | --- | --- | --- |
|  | χ_SB_^2^ | df | CFI | SRMR | RMSEA [90% CI] |  | Models | Δχ_SB_^2^ | ΔCFI | ΔRMSEA |
| Prejudice (ethnic majority participants only) | | | | | | | | | | |
| Configural (M1) | 1013.381 | 672 | .983 | .029 | .022 [.020, .025] |  |  |  |  |  |
| Metric (M2) | 1061.253 | 702 | .982 | .030 | .022 [.020, .025] |  | M2-M1 | 49.749(30)^*^ | -.001 | .000 |
| Scalar (M3) | 1271.855 | 738 | .973 | .034 | .027 [.024, .029] |  | M3-M2 | 285.955(36)^***^ | -.009 | .005 |
| Attitudes toward integration | | | | | | | | | | |
| Configural (M1) | 3052.830 | 1295 | .925 | .047 | .033 [.032, .035] |  |  |  |  |  |
| Metric (M2) | 3159.336 | 1337 | .922 | .052 | .033 [.032, .035] |  | M2-M1 | 108.508(42)^***^ | -.003 | .000 |
| Scalar (M3) | 3540.208 | 1385 | .908 | .064 | .036 [.034, .037] |  | M3-M2 | 444.304(48)^***^ | -.014 | .003 |
| Partial scalar^1^ (M3a) | 3363.725 | 1367 | .914 | .058 | .035 [.033, .036] |  | M3a-M2 | 242.628(30)^***^ | -.008 | .002 |

*Note*. ^1^In this model, the intercept of items 3, 4, and 8 were freed. M = model; χ_SB_^2^ = Satorra-Bentler scaled chi-square; df = degree of freedom; CFI = Comparative Fit Index;

SRMR = Standardized Root Mean Square Residual; RMSEA = Root Mean Square Error of Approximation; CI = confidence interval; Δ = change in the parameter.

^*^ *p* < .05; ^***^ *p* < .001

**Sensitivity analyses: Models with covariates**

A first set of sensitivity analyses was conducted by replicating the multigroup latent growth curve models of prejudice and attitudes toward integration with the inclusion of adolescents’ ethnic background (0 = ethnic majority Italian, 1 = ethnic minority), age, sex (0 = male, 1 = female), and their parents’ educational level (obtained as the sum of mother’s and father’s educational levels) as covariates. In each model, covariates were correlated with growth parameters (intercept and slope(s)). Furthermore, the Wald test was used to examine whether the effect of each covariate significantly differs among participants with intragroup, intergroup, and changing best friendship types. Overall, these sensitivity models fully replicated findings of the main models. Of note, these models were accompanied by warnings about the limited number of clusters compared to the number of model parameters. This also resulted in poor model fit for both the model with prejudice (χ^2^(df) = 1035.044(147), *p* < .001; CFI = .736; RMSEA = .122 [.115, .129]; SRMR = .110) and the one with attitudes towards integration (χ^2^(df) = 1103.411(159), *p* < .001; CFI = .661; RMSEA = .121 [.114, .127]; SRMR = .137). As such, findings should be interpreted with caution. Results of growth parameters are reported in Table S6.

The multigroup model of prejudice including covariates confirmed the differences in intercept and quadratic slopes, although the difference in quadratic slope between the stable intergroup and the changing best friendship types became marginally not significant (*p* = .055). Additionally, a small significant difference emerged between the linear slope of prejudice for adolescents with stable intragroup and those with stable intergroup best friendship (Wald = 3.89, *p* = .049), with the latter (but not the former) showing a significant decrease. Similarly, the difference in the association between opportunity for contact and intercept between adolescents with changing and those with a stable intragroup best friend lost significance (*p* = .349). For what concerns the role of covariates, a few differences emerged. Ethnic minority adolescents with a changing friendship type, but not those with a stable intragroup best friend (Wald = 6.36, *p* = .012), reported lower initial levels of prejudice (*r* = -.51, *p* < .001). Further, female participants with a stable intragroup friendship, but not those with a changing friendship (Wald = 5.48, *p* = .019), reported lower initial levels of prejudice (*r* = -.24, *p* < .001).

Similarly to the model without covariates, the multigroup model of attitudes towards integration including covariates did not indicate any significant difference in intercept and slope parameters. However, a significant difference emerged in the association between structural opportunity and rates of change between adolescents with a stable intragroup and those with a stable intergroup best friendship type (Wald = 6.02, *p* = .014). Specifically, only for the latter, more opportunity for contact with the outgroup in the classroom was linked to a steeper decrease in attitudes towards integration. For what concerns the role of covariates, a few differences emerged among groups for the associations between covariates and the slope. Ethnic minority participants with a stable intergroup friendship, but not those with a changing friendship (Wald = 6.66, *p* = .010), reported a less steep decrease in attitudes toward integration (*r* = -.77, *p <*.001). Similarly, adolescents whose parents had a higher educational level and had a stable intergroup best friendship, but not those with a stable intragroup (Wald = 5.86, *p* = .015) and a changing best friendship (Wald = 7.03, *p* = .008), displayed a less steep decrease in attitudes towards integration (*r* = -.16, *p* = .042).

**Table S6**

Results of models with covariates

|  | Type of best friendship | | |
| --- | --- | --- | --- |
|  | Stable intragroup | Stable intergroup | Changing best friend |
| **Prejudice** |  |  |  |
| **Growth parameters *M* (*σ^2^*)** |  |  |  |
| Intercept | 4.10_a_^***^  (5.33^***^) | 2.20_c_^***^  (3.23) | 3.24_b_^***^  (6.17^**^) |
| Linear slope | -0.05_b_  (0.69^***^) | -0.43_a_^*^  (0.78) | -0.08_a,b_  (1.16) |
| Quadratic slope | 0.01_b_  (0.02^***^) | 0.08_a_^**^  (0.02) | 0.01_(b)_  (0.02^*^) |
| **Standardized correlations** |  |  |  |
| Opportunity **↔** Intercept | 0.08 | 0.00 | 0.25 |
| Opportunity **↔** Linear slope | -0.06 | -0.03 | -0.39 |
| Opportunity **↔** Quadratic slope | 0.09 | 0.10 | 0.30 |
| **Attitudes toward Integration** |  |  |  |
| **Growth parameters *M* (*σ^2^*)** |  |  |  |
| Intercept | 3.98^***^  (0.26^***^) | 3.98^***^  (0.23^***^) | 4.00^***^  (0.27^***^) |
| Slope | -0.05^***^  (0.00^***^) | -0.03^*^  (0.01) | -0.04^***^  (0.00) |
| **Standardized correlations** |  |  |  |
| Opportunity **↔** Intercept | -0.09 | -0.10 | -0.14 |
| Opportunity **↔ S**lope | -0.11_b_ | 0.67_a_^***^ | 0.20_a,b_ |

*Note*. Subscript letters within the same line indicate significant differences between adolescents in the three groups. Subscript letters in parenthesis indicate that the Wald test was only marginally significant. Grey-shaded values lost significance when accounting for covariates, while blue-shaded values became significant when accounting for covariates.

^*^*p* < .05; ^**^*p* < .01; ^***^*p* < .001

**Sensitivity analyses: Alternative grouping of types of best friendships**

To further nuance our understanding of different trajectories of best friendships, a second set of sensitivity analyses was conducted by relying on an alternative grouping of best friendship types. Specifically, the “changing best friendship” type included adolescents with various trajectories of best friendship types, with some moving from an intragroup to an intergroup best friend, others following the opposite pathway, and others alternating between different types of best friendship across the study occasions. To understand whether these diverse trajectories have a meaningful impact on the development of prejudice and attitudes towards integration, the main latent growth curve models of these two constructs were tested in a multigroup format using an alternative grouping solution with five types of best friendships. Specifically, we distinguished: (0) adolescents with a *stable intragroup best friend* across the study period (70.42% of adolescents), (1) those with a *stable intergroup best friend* throughout the study (11.08% of adolescents), (2) those who *lost an intergroup best friend* (i.e., they started the study with an intergroup best friend but at some point switched to an intragroup best friend; 4.97% of adolescents), (3) those who *gained an intergroup best friend* (i.e., they started the study with an intragroup best friend but at some point switched to an intergroup best friend; 5.05% of adolescents), and (4) those who showed *multiple shifts* across the measurement occasions (e.g., they started with an intragroup, moved to an intergroup, and then back to an intragroup best friend; 8.48% of adolescents).

The multigroup latent growth curve model of prejudice, controlling for structural opportunity for contact in the classroom, showed a good fit: χ^2^(df) = 237.396(115), *p* < .001; CFI = .957; RMSEA [90% C.I.] = .066 [.054, .078]; SRMR = .057. Results are reported in Table S7 and displayed in Figure S1. Several differences emerged among the five groups of adolescents only in terms of growth parameters. Regarding intercept scores, in line with the main analyses, adolescents with a stable intragroup best friendship displayed the highest initial levels of prejudice compared to those with a stable intergroup best friend (Wald = 34.21, *p* < .001), those who lost an intergroup best friend (Wald = 5.79, *p* = .017), those who gained an intergroup best friend (Wald = 9.73, *p* = .002), and those who shifted multiple times across best friendship types (Wald = 6.92, *p* = .009). Additionally, adolescents with a stable intergroup best friendship reported lower initial levels of prejudice compared to those who lost an intergroup best friend (Wald = 4.16, *p* = .041) and those who shifted across various best friendship types (Wald = 8.62, *p* = .003), but they did not differ from adolescents who gained an intergroup best friend over the course of the study (Wald = 3.35, *p* = .067). Regarding the linear slope score, only a marginally significant difference emerged between adolescents with a stable intragroup and those with a stable intergroup best friendship type (Wald = 3.74, *p* = .053), with the latter showing a significant linear decrease in prejudice over time. Similarly, adolescents with a stable intergroup best friendship differed from those with a stable intragroup (Wald = 4.24, *p* = .039) and from those who gained an intergroup best friend over time (Wald = 5.85, *p* = .015), with the former showing a significant quadratic decrease in prejudice. No significant differences emerged in the associations between structural opportunity for contact in the classroom and growth parameters. All in all, results of these alternative grouping of types of best friendships replicated most of the main findings and highlighted additional nuances in the experiences of adolescents. However, the three groups that were derived from the “changing best friendship” type, namely the lost intergroup, gained intergroup, and multiple shifts groups, did not significantly differ from each other. This means that relying on the grouping with three types of best friendships provides a more parsimonious representation of adolescents’ experiences.

When using the alternative grouping solution, the multigroup latent growth curve model of attitudes towards integration did not converge. This was possibly due to the small number of observations in some of the categories of best friendship and the complexity of the model. As such, we examined possible group differences in attitudes towards integration across the study occasions by testing seven univariate ANOVAs (i.e., one for each study assessment) in SPSS. Results highlighted a small, albeit significant difference among the five types of best friendship in initial levels of attitudes towards integration (*F*(df) = 2.434(4), *p* = .046, η^2^ = .011). Post-hoc comparisons highlighted that adolescents with a stable intergroup and those with multiple shifts in best friendship type displayed significantly more positive attitudes towards integration at the beginning of the study compared to adolescents who lost an intergroup best friendship. No significant differences emerged at all the other time occasions (T2: *F* = 1.419(4), *p* = .226, η^2^ = .006; T3: *F* = 0.289(4), *p* = .885, η^2^ = .001; T4: *F* = 0.147(4), *p* = .964, η^2^ = .001; T5: *F* = 1.030(4), *p* = .391, η^2^ = .005; T6: *F* = 0.670(4), *p* = .613, η^2^ = .004; T7: *F* = 0.802(4), *p* = .524, η^2^ = .004), suggesting that different types of best friendship had no influence on the development of attitudes.

**Table S7**

Results of models with alternative groups

|  | Types of best friendships | | | | |
| --- | --- | --- | --- | --- | --- |
|  | Stable intragroup | Stable intergroup | Lost intergroup | Gained intergroup | Multiple  shifts |
| **Prejudice** |  |  |  |  |  |
| **Growth parameters *M* (*σ^2^*)** |  |  |  |  |  |
| Intercept | 4.09_a_^***^  (5.26^***^) | 2.18_c_^***^  (3.04^*^) | 3.15_b_^***^  (6.98^***^) | 2.96_b, c_^***^  (5.28^***^) | 3.30_b_^***^  (4.46^***^) |
| Linear slope | -0.04_(b)_  (0.68^***^) | -0.43_(a)_^*^  (0.72) | 0.06_a, b_  (2.06) | -0.08_a, b_  (0.43) | -0.10_a, b_  (0.91) |
| Quadratic slope | 0.01_b_  (0.02^***^) | 0.08_a_^**^  (0.02) | -0.01_a, b_  (0.05) | -0.01_b_  (0.01) | 0.02_a, b_  (0.03) |
| **Standardized correlations** |  |  |  |  |  |
| Opportunity **↔** Intercept | -0.02 | -0.27^***^ | -0.43^*^ | -0.30 | -0.23^*^ |
| Opportunity **↔** Linear slope | -0.05 | 0.08 | -0.02 | 0.12 | -0.10 |
| Opportunity **↔** Quadratic slope | 0.05 | -0.10 | 0.00 | -0.11 | 0.09 |
| **Attitudes towards integration^1^** |  |  |  |  |  |
| Time 1 *M*(*SD*) | 4.02_a, b_ (0.73) | 4.14_a_  (0.64) | 3.80_b_  (0.89) | 3.96_a, b_  (0.78) | 4.17_a_  (0.60) |
| Time 2 *M*(*SD*) | 3.97  (0.66) | 4.14  (0.58) | 4.02  (0.57) | 3.98  (0.56) | 4.01  (0.66) |
| Time 3 *M*(*SD*) | 3.90  (0.68) | 3.94  (0.67) | 4.01  (0.68) | 3.86  (0.64) | 3.92  (0.66) |
| Time 4 *M*(*SD*) | 3.81  (0.72) | 3.86  (0.69) | 3.80  (0.64) | 3.76  (0.61) | 3.83  (0.75) |
| Time 5 *M*(*SD*) | 3.78  (0.71) | 3.94  (0.74) | 3.75  (0.53) | 3.88  (0.66) | 3.80  (0.70) |
| Time 6 *M*(*SD*) | 3.76  (0.73) | 3.83  (0.78) | 3.64  (0.80) | 3.78  (0.72) | 3.67  (0.74) |
| Time 7 *M*(*SD*) | 3.77  (0.73) | 3.79  (0.78) | 3.77  (0.81) | 4.00  (0.60) | 3.82  (0.71) |

*Note*. Subscript letters within the same line indicate significant differences between adolescents in the five groups based on Wald test (for prejudice) and Tukey post-hoc comparisons (for attitudes towards integration). Subscript letters in parentheses indicate that the Wald test was only marginally significant.

^1^The multigroup latent growth curve model of attitudes towards integration did not converge. Seven univariate ANOVAs were tested to examine differences in means and standard deviations of attitudes towards integration at each study occasion.

^*^*p* < .05; ^**^*p* < .01; ^***^*p* < .001

**Figure S1**

Development of prejudice across five types of best friendships


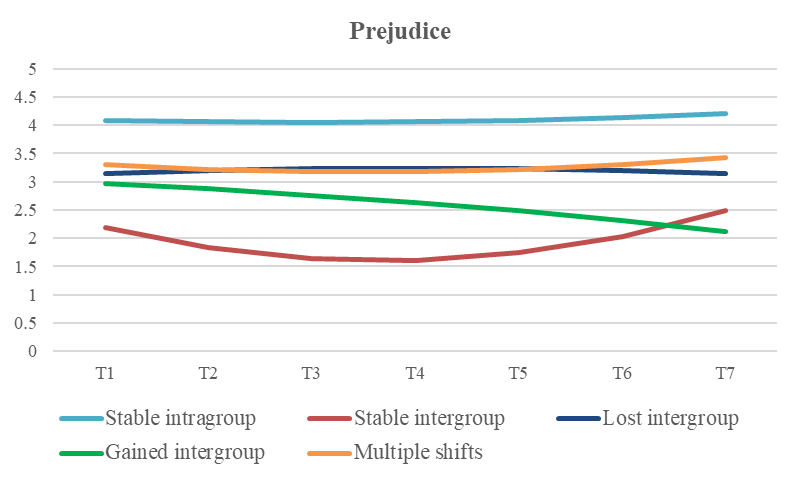


*Note.* Prejudice was assessed on a scale from 0 to 10 but the assessment scale in the figure was adjusted to facilitate the interpretation of findings.
